# Differences in Psychosocial Protective Factors by Race/Ethnicity and Socioeconomic Status and Their Relationship to Preterm Delivery

**DOI:** 10.1089/whr.2021.0049

**Published:** 2022-02-28

**Authors:** Yasamean Zamani-Hank, Claire E. Margerison, Nicole M. Talge, Claudia Holzman

**Affiliations:** Department of Epidemiology and Biostatistics, College of Human Medicine, Michigan State University, East Lansing, Michigan, USA.

**Keywords:** health disparities, pregnancy, preterm delivery, protective factors, race/ethnicity, socioeconomic status

## Abstract

***Background:*** Non-Hispanic Black (“Black”) women in the United States deliver preterm at persistently higher rates than non-Hispanic White (“White”) women, and disparities in preterm delivery (PTD) also exist by socioeconomic factors. Research is needed to identify and understand factors that are protective against PTD for Black women and low socioeconomic status (SES) women.

***Methods:*** We examined seven potential protective factors at the individual, interpersonal, and neighborhood levels during pregnancy to determine if they (1) differed in prevalence by race/ethnicity and SES and (2) were associated with risk of PTD overall or within specific race/ethnicity and SES groups. We used prospectively collected data from *n* = 2474 women who were enrolled in the Pregnancy Outcomes and Community Health Study conducted in Michigan (1998–2004).

***Results:*** White women reported higher levels of self-esteem, mastery, perceived social support, instrumental social support, and reciprocity compared to Black women (all *p* < 0.01), while Black women reported higher levels of religiosity compared to white women (*p* < 0.01). High SES women reported higher levels of all protective factors compared to middle and low SES women (all *p* < 0.01). While protective factors were not independently associated with PTD, religiosity was associated with lower odds of PTD among low SES women (OR 0.6, 95% CI 0.4-0.9) and among Black women (OR 0.6, 95% CI 0.4–1.0), respectively.

***Conclusions:*** Our findings highlight the importance of assessing how protective factors may operate differently across race/ethnicity and SES to promote healthy pregnancy outcomes. Future studies should examine mechanisms that elucidate potential causal pathways between religiosity and PTD for Black women and low SES women.

## Introduction

### Disparities in preterm delivery

Although non-Hispanic black women (hereafter, “Black”) in the United States exhibit persistently higher rates of preterm delivery (PTD) than non-Hispanic white (hereafter, “White”) women, the majority of Black women (86%) do not have PTDs.^1^ Research has focused intensively on studying the factors that increase the risk of PTD among the 14% of Black women who deliver preterm.^2–8^

Hypothesized explanations for high rates of PTD in Black women include social stressors (*e.g.*, stressful life events, racial discrimination, and adverse childhood experiences), psychological factors (*e.g.*, anxiety and depression), health behaviors (*e.g.*, nutrition and substance use), and biological dysregulation (*e.g.*, inflammation).^3,5,9,10^ However, these factors have not satisfactorily “explained” racial/ethnic disparities in PTD.^9,11^ Unacceptable Black-White disparities in PTD persist and have not changed significantly in nearly 30 years.^1,12^

In addition to race/ethnicity, disparities in PTD also exist by socioeconomic factors. Individual-level occupational class and aggregate socioeconomic measures based on census tract (poverty, income, education, unemployment, and wealth) are consistently associated with increased risk of preterm birth.^13^ Moreover, census-based socioeconomic measures (*e.g.*, neighborhood poverty and unemployment) are consistently linked to increased risk of preterm birth among Black women, but not consistently so among White women.^14–19^

Racial disparities in PTD also exist by individual-level socioeconomic measures.^16–18^ For example, Black women with less than a high school education exhibited increased odds of spontaneous PTD (odds ratio [OR] = 2.34, confidence interval [95% CI] 1.15–4.74) relative to women who completed high school, but this finding was not observed among White women with less than a high school education.^17^ Together, these findings suggest that disparities in adverse birth outcomes like PTD occur along dimensions of both race/ethnicity and socioeconomic status (SES).

### Protective factors and health outcomes

Black women and low SES women often have greater exposure to an array of adverse life experiences due to “multiple marginalizations” at the intersection of race, sex/gender, and socioeconomic position.^20^ While dismantling the systemic contributions to adversity are critical to creating equity in pregnancy health and birth outcomes, understanding the protective factors that promote healthy pregnancy outcomes for women in “multiply marginalized”^20^ positions in society may provide useful targets for intervention.

Protective factors include resources people can tap into for strength and support to overcome adversities and significant life stressors.^21–25^ Protective factors exist at multiple socioecological levels and are context specific.^21,23,26–29^ At the individual level, such protective factors might be self-esteem, self-efficacy, internal locus of control, hopefulness, mastery, spirituality, social attachment, optimism, resourcefulness, and flexibility.^23,26,30^ At the interpersonal level, protective factors may include social support, close relationships, cohesion with family and friends, social support from mentors such as teachers, and marital support.^23,26,30^ Community-level factors may include neighborhood and community support, social cohesion, reciprocal exchange, culture, and beliefs in societal values.^23,24,26,30^

Among adults, specific protective factors such as social support have been associated with multiple health outcomes, including decreased risk of depression,^31^ self-efficacy in relation to substance use recovery,^32^ improved weight loss outcomes for obesity,^33^ improved glycemic control in diabetes,^34^ smoking cessation,^35^ and greater adherence to antiretroviral therapy for HIV.^36^ Having low levels of social networks has been associated with increased risk of stroke and cardiovascular mortality,^37^ while low functional social support has been associated with higher risk of mortality among patients with coronary heart disease.^38^

Similarly, religiosity and spirituality have been associated with improved quality of life among patients with cardiovascular disease,^39^ lower blood pressure,^40^ fewer symptoms of depression,^41,42^ and better management of diabetes.^43,44^

Availability and use of specific protective factors appear to differ by gender, race/ethnicity, and SES. For example, women are more likely to seek social support and report higher religiosity compared to men.^45–47^ Black individuals, particularly Black women, report greater religious involvement.^47–49^ Among low SES individuals, protective factors such as religiosity, high levels of perceived control, having a purpose in life, and high optimism have been identified as promoting positive health outcomes in the face of adversity.^50,51^ These findings provide a basis for investigating whether and how protective factors influence disparities in adverse pregnancy outcomes by race/ethnicity and SES.

### Protective factors and birth outcomes

There is increased attention to studying how protective factors, particularly social support, may either (1) directly reduce risk of adverse pregnancy outcomes or (2) buffer against negative impacts on pregnancy outcomes of factors such as adverse childhood experiences, perceived stress, and stressful life events.^52–55^

For example, among female teenagers enrolled in a WIC program who had uncomplicated healthy pregnancies, factors at both the individual level (positive outlook, self-efficacy, and prenatal care attendance) and family level (parental and partner social support) were identified as protective influences, emphasizing the role that such factors may play in contributing to resilient pregnancy outcomes among low-SES women.^25^ However, in a meta-analysis, investigators noted no evidence of a direct association between social support and preterm birth based on the pooled findings of eight studies.^52^

On the other hand, pooled findings from two studies which examined the buffering effects of social support found that women with low social support and high stress levels experienced higher odds of preterm birth compared to women with high social support and high stress levels.^52^ Specifically, women with high levels of cumulative psychosocial stress (operationalized by “state anxiety” and history of mental health problems, abuse, and negative feelings about pregnancy timing) and low levels of perceived social support experienced higher odds of preterm birth (OR 2.09, 95% CI 1.07–4.07).^54^

The second study examined multilevel protective factors such as self-esteem, mastery, partner support, social support, and neighborhood support and found that women with higher levels of stress compared to protective factors (as operationalized by a higher stress-to-capital ratio [SCR]) had higher odds of experiencing premature labor compared to women who had lower SCRs (OR = 1.36; *p* = 0.03).^30^ Furthermore, Black and Hispanic women had higher mean SCR scores compared to White women (*p* < 0.001, respectively), while low-income women had higher SCR scores compared to higher-income women.

Together, these findings suggest that protective factors may reduce the risk of adverse pregnancy outcomes in the context of stress, and that protective factors may differ by race/ethnicity and SES. While providing significant contributions to an understanding of protective factors in relation to pregnancy outcomes, previous studies have (1) focused primarily on social support and rarely incorporated data on additional protective factors at multiple socioecological levels and (2) typically not assessed how protective factors vary by race/ethnicity and SES.

Understanding the psychosocial factors that influence pregnancy outcomes may lend useful insight in clinical settings to help women “identify, affirm, and build” (Walsh, p. 140)^21^ their repertoire of individual, interpersonal, and community factors, which can be mobilized to promote a healthy pregnancy outcome. Thus, the objectives of our research study were to examine potential protective factors at multiple socioecological levels (individual, interpersonal, and neighborhood) during pregnancy and determine if they (1) differ in prevalence by race/ethnicity and SES and (2) are associated with risk of PTD overall or within specific race/ethnicity and SES groups.

## Methods

### Study design, dataset, and study population

We utilized data from the Pregnancy Outcomes and Community Health (POUCH) study, a prospective cohort investigation of pregnant women (*n* = 3019) from five diverse communities across Michigan between 1998 and 2004, with the primary goal of understanding physiological and psychosocial risk factors for PTD. Fifty-two clinics from Lansing, Kalamazoo, Saginaw, Flint, and Grand Rapids were invited to participate if their patients delivered at one of eight designated Michigan hospitals in these regions and if they submitted maternal samples to designated Michigan prenatal screening programs for analysis.

Using a stratified random sampling scheme that oversampled Black women and pregnant women with unexplained high maternal serum alpha-fetoprotein (MSAFP) during screening (a risk factor for PTD), pregnant women were invited to participate if they were English-speaking and older than 14 years of age and if they received MSAFP screening during 15–20 weeks of gestation, had a singleton pregnancy with no congenital or chromosomal abnormalities, and had no history of diabetes mellitus. Details about the study design and protocols have been described previously.^56,57^

**FIG. 1.**
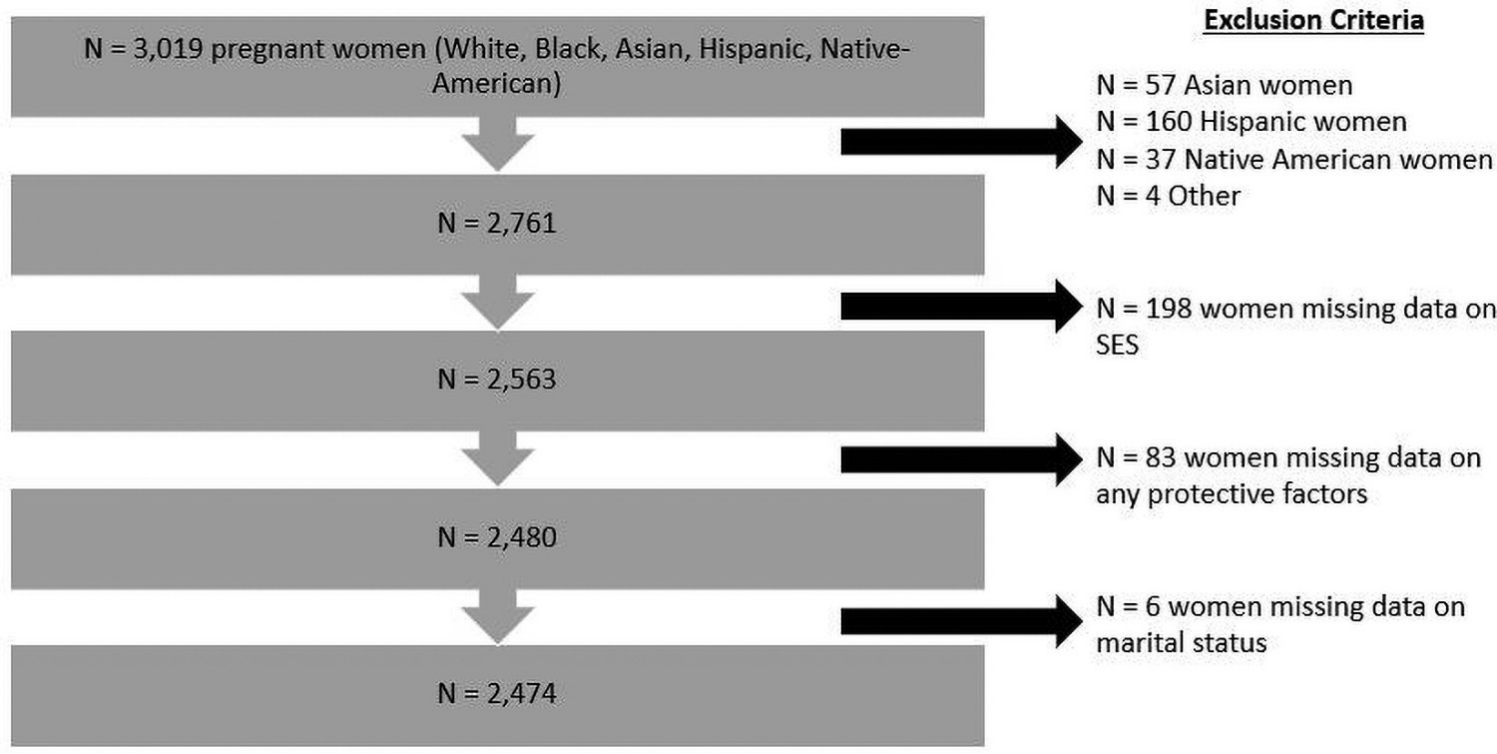
Analytic sample used to assess association between PTD and seven multilevel protective factors among pregnant women in the Pregnancy Outcomes and Community Health Study (1998–2004). PTD, preterm delivery.

Of the 3019 enrolled pregnant women in the POUCH Study, we excluded women from racial/ethnic groups with small sample sizes (Asian, Hispanic, Native-American, and Other) and women with missing data on sociodemographics, protective factors, or marital status to establish our analytic sample of *n* = 2474 Black and White women (Fig. 1). The POUCH Study was approved by the institutional review boards at Michigan State University, Michigan Department of Community Health, and nine community hospitals.

### Measures

*Race/ethnicity* (non-Hispanic White, non-Hispanic Black, hereafter “White” and “Black”) was collected through self-report on POUCH Study questionnaires. We recognize that “race is a social construct”^58^ (p. 303) and has no biological basis,^59,60^ although race has real-life impacts on the health outcomes of individuals.^59,61^ In our study, race refers to the social classification process by which individuals are categorized into groups based on their phenotype within racially stratified societies.^58^

#### Protective factors

The primary exposure variables we examined were seven psychosocial^62^ protective factors at the individual (self-esteem, mastery, and religiosity), interpersonal (perceived social support, instrumental social support, and emotional social support), and neighborhood (reciprocity) levels.

We asked women about seven types of protective factors at study enrollment (16–27 weeks of gestation) using self-administered questionnaires. These factors included: perceived social support (Multidimensional Scale of Perceived Social Support^63^), emotional social support (Strogatz Social Support^64^), instrumental social support (Strogatz Social Support^64^), self-esteem (Rosenberg Self-Esteem Scale^65^), mastery (Pearlin Mastery Scale^66^), reciprocity (developed by the POUCH Study team), and religiosity (developed by the POUCH Study team).

The perceived social support subscale consisted of 3 items (*α* = 0.70), the instrumental social support subscale consisted of 3 items (*α* = 0.44), the mastery subscale consisted of 7 items (*α* = 0.53), the self-esteem subscale consisted of 10 items (*α* = 0.88), and the reciprocity subscale consisted of 3 items (*α* = 0.86).

We analyzed all protective factors as continuous variables, with the exception of religiosity and emotional social support, which were modeled categorically (yes/no). Religiosity was captured by a single question and refers to women who reported turning to religion as a source of comfort in rough times. Similarly, emotional social support was captured by a single question and refers to women who reported that they have someone to turn to if they are worried about an important personal matter. Instrumental social support refers to help with material needs such as borrowing money, transportation, and house cleaning and repairs.^64^

Reciprocity was captured as a composite measure of three questions, which assessed mutual neighborhood social interactions (visiting and chatting) and assistance with lending tools, transportation, and watching each other's houses. Supplementary Appendix Table SA1 provides detail on all protective factors and survey instruments.

#### Preterm delivery

The primary outcome variable was PTD, defined as a birth before 37 weeks of gestation. Gestational age at delivery was calculated using date of delivery and last menstrual period (LMP) date abstracted from medical records.^57^ If a discrepancy of >2 weeks existed between the date of the LMP and early ultrasound-based estimates of gestational age, the ultrasound data were given preference due to demonstrated greater accuracy of ultrasound compared to LMP-based estimates.^67,68^ For 19% of women, the ultrasound-based estimate was used either because there was a discrepancy of >2 weeks between the ultrasound-based and the LMP-based estimates *or* because there was no LMP date provided in the medical chart and women could not recall the LMP date.

#### Covariates

Study covariates included maternal age (<20, 20–29 [reference], 30–34, and ≥35 years), marital status (married [reference] and not married), and parity (0 [reference], 1, and ≥2 live births) collected through self-report.

SES at study enrollment (low, middle, and high [reference]) was derived by summing six different dichotomized SES measures (woman's education, father-of-baby's education, woman's occupation, father-of-baby's occupation, mother's Medicaid status, and mother's annual household income) into a composite score that ranged from 0 to 6.^69^ Women were categorized into three class groups based on quartiles from the distribution of this score (low SES = bottom quartile, score = 0; middle SES = second and third quartiles, score = 1–3; and high SES = top quartile, score ≥4).^69^

**FIG. 2.**
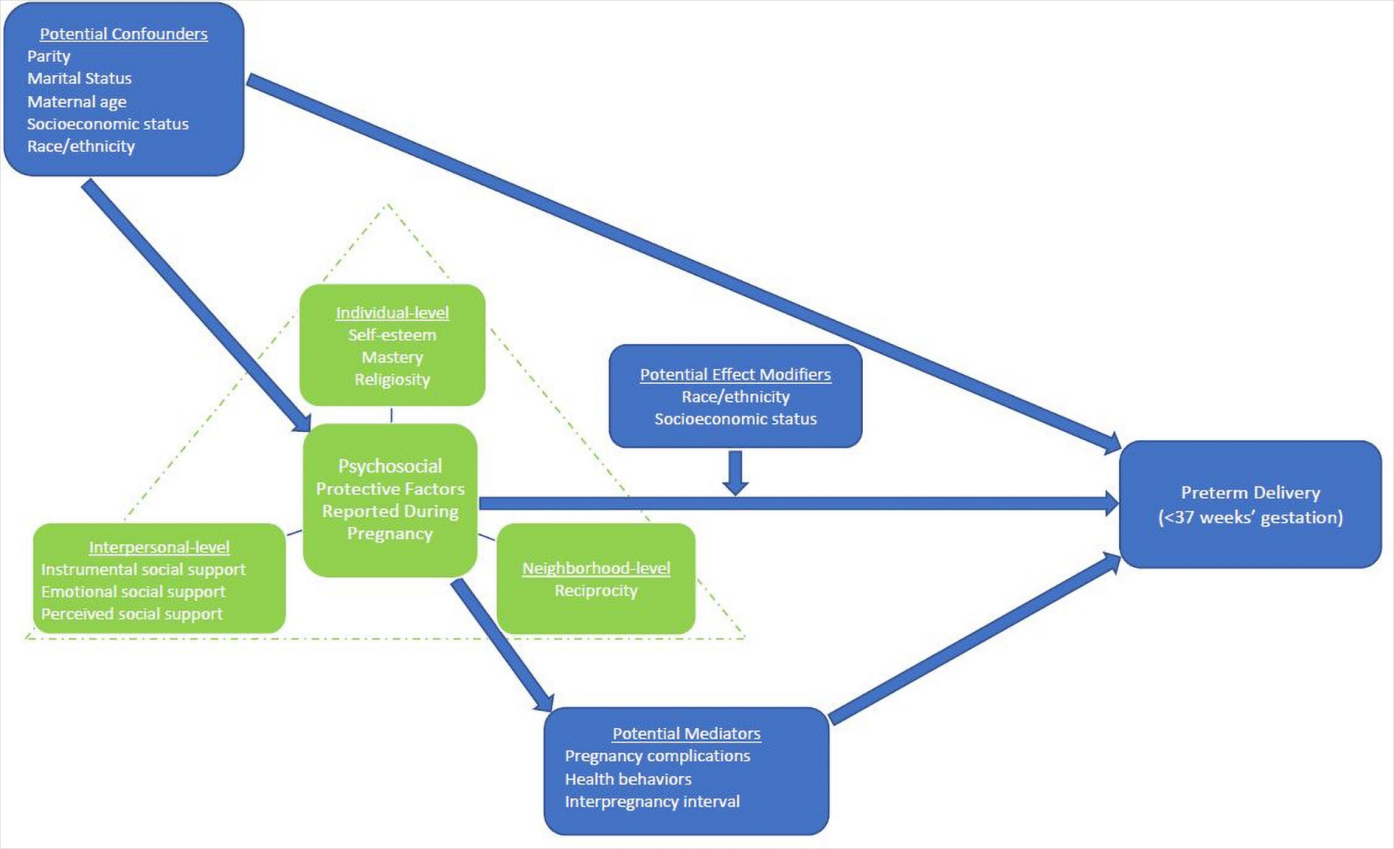
Conceptual diagram of the hypothesized relationship between multilevel psychosocial factors and preterm delivery among pregnant women in the Pregnancy Outcomes and Community Health Study (1998–2004).

Women with missing data on any of these variables were excluded from the analytic sample (Fig. 1). A conceptual diagram of the hypothesized relationships and pathways between protective factors, PTD, and covariates is provided in the appendix (Fig. 2).

### Statistical analyses

We weighted all analyses for the stratified sampling structure of the POUCH study.

#### Correlation analyses

We evaluated the extent to which the seven protective factors were interrelated and observed weak associations (Supplementary Appendix Table SA2). This suggests that these variables may measure different constructs, and thus justified our examination of each protective factor individually, as opposed to using a composite score.

#### Descriptive analyses

We conducted survey-weighted frequency procedures to obtain the sample size and weighted percentages for race/ethnicity, SES, marital status, maternal age at enrollment, parity, and PTD (Table 1).

**Table 1.** Descriptive Characteristics of the Analytic Sample in the Pregnancy Outcomes and Community Health Study, 1998–2004 (*n* = 2474)

	*n* (Weighted %)^a^
Overall	2474
Race/ethnicity	
White	1834 (75.4)
Black	640 (24.6)
SES^b^	
High	737 (30.0)
Middle	1063 (43.0)
Low	674 (26.9)
Marital status	
Married	1307 (53.2)
Not married	1167 (46.8)
Maternal age at enrollment (years)	
<20	332 (13.3)
20–29	1409 (57.2)
30–34	524 (21.1)
≥35	209 (8.4)
Parity^c^	
0	1037 (42.1)
1	833 (33.6)
≥2	604 (24.3)
PTD	269 (10.7)

Results are weighted to account for the stratified sampling structure of the POUCH study.

^a^ Total percentages may not add up to 100% due to rounding.

^b^ SES: composite score of six variables, including mother's education, father's education, mother's occupation, father's occupation, household income, and Medicaid insurance status.

^c^ Parity: number of pregnancies ending in a live birth.

PTD, preterm delivery; SES, socioeconomic status.

#### Regression analyses

For protective factors measured as continuous variables, we performed survey-weighted linear regression models to assess whether each factor differed by race/ethnicity and SES (two tailed, *α* = 0.05) (Table 2). We then used Tukey multiple comparison *post hoc* tests to assess differences in means and estimated the associated effect sizes by calculating Hedges's *g* (0.2 = small, 0.5 = medium, and 0.8 = large).^70,71^

**Table 2.** Descriptive Characteristics of Protective Factors and Preterm Delivery by Race/Ethnicity and Socioeconomic Status in the Pregnancy Outcomes and Community Health Study, 1998–2004 (*n* = 2474)

	All women	White (*n* = 1834)	Black (*n* = 640)		High SES (*n* = 737)	Middle SES (*n* = 1063)	Low SES (*n* = 674)	
Protective factors								
		Mean (SE)		*p* ^a^		Mean (SE)		*p* ^a^
Individual level								
Self-esteem	6.0 (0.0)	6.0 (0.0)	5.8 (0.1)	**<0.01**	6.4 (0.0)	5.9 (0.04)	5.6 (0.1)	**<0.01**
Mastery	21.6 (0.1)	21.7 (0.1)	21.2 (0.1)	**<0.01**	22.6 (0.1)	21.5 (0.1)	20.5 (0.1)	**<0.01**
Interpersonal level								
Perceived social support	10.1 (0.0)	10.3 (0.0)	9.6 (0.1)	**<0.01**	10.7 (0.1)	10.1 (0.1)	9.6 (0.1)	**<0.01**
Instrumental social support	5.7 (0.0)	5.8 (0.0)	5.5 (0.0)	**<0.01**	5.9 (0.0)	5.7 (0.0)	5.5 (0.0)	**<0.01**
Neighborhood level								
Reciprocity	6.9 (0.1)	7.1 (0.1)	6.2 (0.1)	**<0.01**	7.5 (0.1)	6.7 (0.1)	6.5 (0.1)	**<0.01**
	*n* (weighted %)			*p* ^b^	*n* (weighted %)			*p* ^b^
Individual-level								
Religiosity^c^	1504 (60.6)	1044 (56.9)	460 (71.7)	**<0.01**	494 (66.7)	652 (61.3)	358 (52.6)	**<0.01**
Interpersonal level								
Emotional social support^d^	2380 (96.2)	1785 (97.3)	595 (92.8)	**<0.01**	726 (98.5)	1030 (96.9)	624 (92.5)	**<0.01**
PTD								
	*n* (weighted %)			*p* ^e^	*n* (weighted %)			*p* ^b^
Yes	269 (10.7)	176 (9.5)	93 (14.3)	**<0.01**	66 (8.9)	120 (11.2)	83 (12.0)	**0.08**

Results are weighted to account for the stratified sampling structure of the POUCH study. Bolded *p*-values indicate statistical significance at *p* < 0.01.

^a^ *p*-Value for survey-weighted ANOVA.

^b^ *p*-Value for survey-weighted Wald chi-square test.

^c^ Refers to proportion of women who responded “yes” to “Do you turn to religion as a source of comfort in rough times?”

^d^ Refers to proportion of women who responded “yes” to “If you are worried about an important personal matter, is there someone you can go to?”

^e^ *p*-Value for survey-weighted likelihood ratio test.

SE standard error.

For protective factors modeled as categorical variables (religiosity and emotional social support), we performed survey-weighted chi-square tests to assess whether these factors differed by race/ethnicity and SES (two tailed, *α* = 0.05) (Table 2) and calculated associated effect sizes using Cramer's *V* (0.1 = small, 0.3 = medium, and 0.5 = large for 1 degree of freedom; 0.07 = small, 0.21 = medium, and 0.35 = large for 2 degrees of freedom).^71,72^

Next we assessed differences in the prevalence of PTD by race/ethnicity and SES using survey-weighted chi-square and survey-weighted logistic regression with a likelihood ratio test, respectively (Table 2). We used unadjusted and adjusted logistic regression models to evaluate associations between each separate protective factor and odds of PTD (Models 1–2, Table 3). Adjusted models controlled for maternal age, marital status, parity, SES, and race/ethnicity. We also conducted unadjusted and adjusted logistic regression models, which considered associations between all protective factors combined and odds of PTD (Supplementary Appendix Table SA3).

**Table 3.** Associations Between Seven Protective Factors and Odds of Preterm Delivery Assessed in Separate Logistic Regression Models in the Pregnancy Outcomes and Community Health Study, 1998–2004 (*n* = 2474)

Protective factors	Model 1^a^	Model 2^b^	Model 3^c^	Model 4^d^
	OR (95% CI)	OR (95% CI)	Race interaction *p*^e^	SES interaction *p*^e^
Individual level				
Self-esteem	1.0 (0.9–1.1)	1.0 (0.9–1.2)	0.14	1.00
Mastery	1.0 (0.9–1.0)	1.0 (0.9–1.0)	0.18	0.62
Religiosity	0.9 (0.7–1.2)	0.9 (0.7–1.2)	**0.08**	**0.07**
Interpersonal level				
Perceived social support	1.0 (0.9–1.1)	1.0 (0.9–1.1)	0.47	**0.05**
Emotional social support	0.7 (0.4–1.2)	0.8 (0.4–1.4)	0.89	**<0.01**
Instrumental social support	1.0 (0.8–1.2)	1.1 (0.9–1.4)	0.87	0.78
Neighborhood level				
Reciprocity	**1.0 (0.9**–**1.0)**	**1.0 (1.0**–**1.1)**	**0.84**	**0.06**

Results are weighted to account for the stratified sampling structure of the POUCH study; for continuous variables (self-esteem, mastery, perceived social support, instrumental social support, and reciprocity), the OR represents the odds of PTD among women who have higher levels of the protective factor compared to women who have lower levels. For categorical variables (religiosity and emotional social support), the OR represents the odds of PTD among women who responded “yes” compared to women who responded “no.” Bolded *p*-values indicate statistical significance at *p* < 0.10.

^a^ Model 1: unadjusted with protective factors assessed separately.

^b^ Model 2: Model 1 + race/ethnicity, SES, maternal age, parity, and marital status.

^c^ Model 3: Model 2 + interaction term between protective factor and race/ethnicity.

^d^ Model 4: Model 2 + interaction term between protective factor and SES.

^e^ *p*-Values for the maximum likelihood estimate for the interaction term.

CI, confidence interval; OR, odds ratio.

Finally, we looked at potential interactions of each protective factor with race/ethnicity and SES in separate logistic regression models to determine whether the association between each protective factor and PTD differed by race/ethnicity and/or SES (Models 3 and 4, Table 3). For interaction terms in Table 3 with a *p*-value of <0.10, we then calculated the association between that protective factor and odds of PTD separately by race/ethnicity and SES (Table 4). We selected white women as the reference group for race/ethnicity and high SES women as the reference group for SES as these women represent the most privileged groups in society. Statistical analyses were conducted using SAS statistical software package 9.4.

**Table 4.** Associations Between Protective Factors and Odds of Preterm Delivery That Differed Significantly by Race/Ethnicity and Socioeconomic Status (Table 3) Assessed in Separate Logistic Regression Models in the Pregnancy Outcomes and Community Health Study, 1998–2004 (*n* = 2474)

Protective factors	Race/ethnicity (reference = White)		SES (reference = high)		
	Model 3^a^		Model 4^b^		
	White (*n* = 1834)	Black (*n* = 640)	High (*n* = 737)	Middle (*n* = 1063)	Low (*n* = 674)
Individual level	OR (95% CI)		OR (95% CI)		
Religiosity	1.0 (0.7–1.4)	0.6 (0.4–1.0)	0.8 (0.5–1.5)	1.2 (0.8–1.9)	0.6 (0.4–0.9)
Interpersonal level					
Perceived social support	—	—	1.1 (0.9–1.3)	1.0 (1.0–1.2)	0.9 (0.8–1.0)
Emotional social support	—	—	N/A^c^	0.8 (0.3–2.2)	0.6 (0.3–1.3)
Neighborhood level					
Reciprocity	—	—	1.0 (0.9–1.1)	1.1 (1.0–1.1)	0.9 (0.8–1.0)

Adjusted odds ratios shown are derived for protective factors with statistically significant (*p* < 0.10) interaction terms by race/ethnicity or SES as found in Table 3; for continuous variables (perceived social support and reciprocity) the OR represents the odds of PTD among women who have higher levels of the protective factor compared to women who have lower levels. For categorical variables (religiosity and emotional social support), the OR represents the odds of PTD among women who responded “yes” compared to women who responded “no.” Results are weighted to account for the stratified sampling structure of the POUCH study.

^a^ Model 3: unadjusted model + race/ethnicity, maternal age, parity, and marital status + interaction term between protective factor and race/ethnicity.

^b^ Model 4: unadjusted model + race/ethnicity, maternal age, parity, marital status + interaction term between protective factor and SES.

^c^ Cell size inadequate to derive an accurate estimate.

## Results

### Descriptive analyses

The analytic sample (*n* = 2474) comprised 75.4% white women and 24.6% black women (Table 1). More than half of the women in our sample were married (53.2%) and between 20 and 29 years of age (57.2%). The overall occurrence of PTD was 10.7%, and 42.1% of women were nulliparous (Table 1).

### Regression analyses

White women reported significantly higher levels of self-esteem, mastery, perceived social support, emotional social support, instrumental social support, and reciprocity compared to black women (*p* < 0.01, respectively) (Table 2). Black women reported significantly higher levels of religiosity (71.7% vs. 56.9%; *p* < 0.01) compared to white women (Table 2). However, the race/ethnicity differences in the continuous variables were all small in magnitude (Hedges's *g* range 0.1–0.4), except for instrumental social support, which was moderate (Hedges's *g* = 0.5, data not shown).

Differences in emotional social support and religiosity were small in magnitude between white and black women (Cramer's *V* = 0.1, respectively). A significantly higher proportion of black women delivered preterm compared to white women (*p* < 0.01).

All seven protective factors differed significantly by SES (*p* < 0.01, respectively) (Table 2). High SES women (top quartile) reported significantly higher levels of self-esteem, mastery, perceived social support, instrumental social support, and reciprocity compared to both middle SES (second and third quartiles) and low SES (bottom quartile) women (*p* < 0.01, respectively), and middle SES women reported significantly higher levels of all these variables (*p* < 0.01, respectively) compared to low SES women (bottom quartile), with the exception of reciprocity (*p* = 0.13).

While differences in all continuous protective factors between low and middle SES women, and between middle and high SES women, were small in magnitude (Hedges's *g* range 0.1–0.4), the differences between low SES and high SES women were large in magnitude for instrumental social support (Hedges' *g* = 0.8) and moderate for perceived social support (Hedges's *g* = 0.7), mastery (Hedges's *g* = 0.7), and self-esteem (Hedges's *g* = 0.6). Differences in emotional social support and religiosity by SES were small in magnitude (Cramer's *V* = 0.1). There was no statistically significant difference in the proportion of women who experienced PTD by SES (*p* = 0.08) (Table 2).

Among black and white women combined, none of the seven protective factors was significantly associated with odds of PTD in unadjusted or adjusted logistic regression models (Table 3). However, the association between religiosity and PTD differed significantly by race/ethnicity (*p* = 0.08) and SES (*p* = 0.07) (Table 3, Models 3 and 4, respectively). Black women who reported religiosity had decreased odds of PTD compared to black women who did not report religiosity, and this approached significance (OR 0.6, 95% CI 0.4–1.0) (Table 4).

Low SES women who reported religiosity had decreased odds of PTD compared to low SES women who did not report religiosity (OR 0.6, 95% CI 0.4–0.9) (Table 4). The associations between perceived social support, emotional social support, and reciprocity and PTD also differed significantly by SES (*p* = 0.05, *p* < 0.01, and *p* = 0.06, respectively) (Table 3, Model 4).

Low SES women who reported higher perceived social support and reciprocity had decreased odds of PTD compared to low SES women who did not, although this was marginally significant (OR 0.9, 95% CI 0.8–1.0; OR 0.9, 95% CI 0.8–1.0, respectively) (Table 4). The association between emotional social support and PTD was not statistically significant for middle SES (OR 0.8, 95% CI 0.3–2.2) or low SES women (OR 0.6, 95% CI 0.3–1.3) (Table 4).

## Discussion

To elucidate the role that protective factors play in reducing disparities in adverse birth outcomes, we examined whether protective factors at multiple socioecological levels during pregnancy (1) differed by race/ethnicity and SES and (2) were associated with PTD overall or within a specific race/ethnicity or SES group.

### Protective factors, race/ethnicity, and SES

While our study found that white women reported higher levels of all protective factors—with the exception of religiosity—compared to black women during pregnancy, our findings are not consistent with those of Jesse et al., who found that African American women in rural prenatal clinics had higher self-esteem, religiosity, spirituality, and social support compared to Caucasian women.^73^ On the other hand, in a sample from an urban prenatal clinic, Jesse et al. found that African American women had lower self-esteem, higher social support from others, and comparable levels of partner social support compared to Caucasian women.^74^

Discrepancies between our findings and those of previous work may be due, in part, to differences in study population (*e.g.*, urban vs. rural and low income vs. general), sample size, different types of social support assessed, context in which social support is assessed (*e.g.*, coping with a health condition vs. caregiving), and methods of assessing protective factors.^75^

In addition, our findings suggested a gradient effect, whereby high SES women reported the highest levels of protective factors, followed by middle SES women who reported intermediate levels of protective factors, and low SES women who reported the lowest levels of protective factors. Women from lower socioeconomic backgrounds may have fewer social support networks from which to obtain resources, compared to high SES women.^76^

Our finding of higher religiosity among black women compared to white women concurs with literature on the importance of religion as a coping mechanism and source of comfort within the black community, especially for black women.^48,77–80^ Chatters et al. state, “…Religious orientations and strategies are an especially prominent and robust component of the coping repertoires of African Americans who are more likely than their Whites to report their use in response to a variety of problems and contexts including health issues, caregiving burdens, chronic poverty, poor neighborhood conditions, structural exclusion, and interpersonal and structural racism” (p. 373). Moreover, religiosity may be an important source of support for black women, especially in the face of life stressors.^48,49,79,81^

Pregnancy represents a significant period of change for women during which spirituality may play an important role in conferring support and comfort.^82,83^ In our study, although religiosity was asked during pregnancy, it was assessed as a general question, not specific to the prenatal period (Supplementary Appendix Table SA1). Future research using a longitudinal approach is needed to better contextualize the role of religiosity in the lives of reproductive-aged women, including whether findings differ according to race/ethnicity.

### Protective factors and PTD

We found that the specific set of protective factors linked to a reduction in PTD differed by race/ethnicity and SES, although most odds ratios only approached statistical significance. Religiosity was associated with an ∼40% reduction in the odds of PTD for low SES women and black women, consistent with previous studies supporting the protective influence of religiosity on health-related outcomes, including PTD, depression from HIV-related stigma, diabetes, and hypertension, among black women.^49,81,84,85^

Religiosity is also hypothesized to serve as a buffer against the adverse circumstances associated with poverty among low-income populations.^86^ Specifically, “Higher levels of religiosity may provide a pathway out of multi-problem behavioral patterns that can accompany limited resources by promoting better coping mechanisms for economic instability and stress….” (Joshi and Hardy 2009, p. 2). For example, religiosity has been linked previously with reduced risk of smoking, improved positive well-being and health behaviors, self-efficacy, and decreased risk of depression among low-income women.^86^

Religiosity also has been associated with positive health behaviors during pregnancy, including good nutrition, and abstaining from alcohol, smoking, or substance use.^87–90^ Previous POUCH studies also found that women with highest levels of stress hormones during mid-pregnancy had significantly elevated risk of spontaneous PTD compared to women with lowest levels of stress hormones.^57^ Furthermore, women who reported higher levels of hostility and anomie during mid-pregnancy had increased risk of PTD.^91^ Thus, religiosity may protect against the risk of PTD through potential buffering of environmental, physiological, and psychosocial stressors.

Moreover, religiosity has been associated with upward mobility,^92^ another potential pathway between religiosity and lower PTD risk. Women in the POUCH study, who experienced upward socioeconomic mobility from childhood to adulthood, exhibited a lower risk of PTD^93^ as well as delivering a small-for-gestational age infant.^69^ Upward social mobility may serve to decrease a woman's risk of PTD by reducing the body's “wear and tear” in the context of allostatic load^69^ and/or by conferring greater access to certain resources (health care, recreational facilities, and education). Thus, future studies using mediation analyses could better elucidate causal pathways between religiosity and PTD among black women or low SES women.

The findings of previous research on the impact of social support on PTD among low SES women are mixed.^74,94^ Among an urban sample of low-income Black non-Hispanic women, emotional social support was associated with significantly reduced odds of preterm birth, but socializing social support, instrumental social support, and interactive social support were not.^94^ However, among a sample of low-income pregnant women from a rural health clinic, social support was not significantly associated with preterm birth.^74^

Nevertheless, having neighbors who can lend help in times of need, as assessed by neighborhood reciprocity, can contribute to one's perceived levels of social support,^95,96^ and thus influence pregnancy outcomes.^30^ Furthermore, the quality of a neighborhood environment, including safety, social and physical disorder, and walkability, impacts the prevalence of depression and perceived stress levels during pregnancy,^97^ both of which represent risk factors for PTD.^5^ Positive aspects of a neighborhood, including reciprocity and social cohesion, buffer against depression, specifically among women,^98^ highlighting the importance of community-level protective factors on women's health outcomes.

Thus, our finding that reciprocity buffers against PTD among low SES women may suggest possible mediating mechanisms through its impact on pregnant women's mental health and perceived stress levels.

### Strengths and limitations

Strengths of our approach included that we selected protective factors based on interdisciplinary frameworks from ecological theory,^27^ resilience science and developmental psychology,^21,24,28^ and social epidemiology.^99^ We also utilized a sample of women from 52 clinics across 5 diverse communities, whereas prior work on protective factors in pregnancy has sampled from a few clinics within academic or urban referral care settings. Study questionnaires were distributed to study participants to complete on their own (*i.e.*, without supervision of study staff), promoting confidentiality and accuracy.

Our study was limited by sample size considerations that prevented us from including racial/ethnic groups other than White and Black women. Limited cell sizes also prevented us from assessing more than one interaction (*i.e.*, race/ethnicity × protective factor, SES × protective factor) in the same model, as well as exploring subcategories of preterm birth (*i.e.*, early vs. late preterm). In addition, religiosity and emotional social support were only assessed with a single question and do not capture the multidimensional nature of these constructs. Furthermore, as noted earlier, we assessed protective factors only at one time point during pregnancy, instead of utilizing a longitudinal approach to capture the socioecological factors across the life course that may impact pregnancy health outcomes.^11^

## Conclusion

Overall, our findings suggest that the *specific set* of protective factors that buffer against adverse birth outcomes like PTD may differ among women by race/ethnicity and SES, a finding that corroborates previous research.^74^ On the other hand, among all women, we did not find that protective factors at the individual, interpersonal, or neighborhood levels were independently associated with PTD. The “protective” aspect of protective factors on health outcomes may not manifest unless adversity is presented.^24,28^ Indeed, Margerison-Zilko et al. found that certain types of adversity, specially childhood sexual abuse, were associated with increased risk of PTD in the POUCH cohort.^100^

Thus, to comprehensively assess the effects of protective factors on pregnancy outcomes, future research should also incorporate measures of adversity to determine whether protective factors moderate the association between adverse life experiences and PTD risk.

Our findings highlight the need for research, which applies a theoretically grounded framework of protective factors to perinatal health to understand their role in promoting healthy pregnancy and birth outcomes. Identification of new ways to conceptualize and measure protective factors, with careful attention to cultural appropriateness and context, will be essential to this effort. Moreover, in relation to public health and clinical practice, an assessment of the availability of a woman's protective factors during pregnancy can be used to help identify women and families who can be connected with community-based resources.^21^

An understanding of the psychosocial factors must also acknowledge the structural barriers (such as racism, discrimination, access to health care, education, and socioeconomic resources) that may influence a woman's repertoire of and ability to mobilize protective factors at all socioecological levels. In the words of Seccombe, “Without sound policies, individual attributes, involved families, and supportive communities will have limited effectiveness.” (p. 389).^101^ Ultimately, elucidating the influence of protective factors on birth outcomes to multiply marginalized groups of women has implications for effective interventions to mitigate perinatal health disparities by race and SES.

## Supplementary Material

Supplemental data

Supplemental data

Supplemental data

## Supplementary Material

Supplementary Appendix Table SA1

Supplementary Appendix Table SA2

Supplementary Appendix Table SA3

## Abbreviations Used

CI: confidence interval
LMP: last menstrual period
MSAFP: maternal serum alpha-fetoprotein
OR: odds ratio
PF: protective factors
PTD: preterm delivery
SCR: stress-to-capital ratio
SES: socioeconomic status
SGA: small-for-gestational age
