# Supplementary material for: Differences in Psychosocial Protective Factors by Race/Ethnicity and Socioeconomic Status and Their Relationship to Preterm Delivery
Source: Womens Health Rep (New Rochelle). 2022 Feb 28;3(1):243–55. doi: 10.1089/whr.2021.0049 (PMC8896219; doi:10.1089/whr.2021.0049)
Supplement: Supplemental data [file Suppl_AppTableS1.docx]

**Supplementary Appendix**

Table 1. Protective factors collected in the Pregnancy Outcomes and Community Health Study (n = 2,474), 1998-2004.

| Multi-Level Protective Factors | Origin of Scale | Example items in measure | Type of Variable / Possible responses |
| --- | --- | --- | --- |
| *Individual-Level* |  |  |  |
| Self-Esteem | Rosenberg Self-Esteem Scale^65^ | I feel that I’m a person of worth, at least on an equal plane with others. | Continuous (1-7) strongly disagree, disagree, agree, strongly agree |
| Mastery | Pearlin Mastery Scale^66^ | I have little control over the things that happen to me. | Continuous (7-28)  Strongly disagree, disagree, slightly agree, agree, strongly agree |
| Religiosity | Developed by the POUCH Study Team (Reference: Brief Multidimensional Measure of Religiousness/Spirituality^95^) | Do you turn to religion as a source of comfort in rough times? | Yes/No |
| *Interpersonal-Level* |  |  |  |
| Perceived social support | Multidimensional Scale of Perceived Social Support^63^ | My family really tries to help me. | Continuous (3-12) Strongly disagree, disagree, agree, strongly agree |
| Emotional social support | Strogatz Social Support^64^ | If you are worried about an important personal matter, is there someone you can go to? | Yes/No |
| Instrumental social support | Strogatz Social Support^64^ | If you needed to borrow a fairly large sum of money, do you have someone or somewhere you could borrow it from? | Continuous (3 -6) |
| *Neighborhood-Level* |  |  |  |
| Reciprocity | Developed by the POUCH Study Team (Reference: Project on Human Development in Chicago Neighborhoods^90^) | How often do you and your neighbors help each other out by lending things like tools, giving someone a ride, or watching each other’s houses when you’re away? | Continuous (3-12)  Never, seldom, sometimes, often |
