# Supplementary material for: Differences in Psychosocial Protective Factors by Race/Ethnicity and Socioeconomic Status and Their Relationship to Preterm Delivery
Source: Womens Health Rep (New Rochelle). 2022 Feb 28;3(1):243–55. doi: 10.1089/whr.2021.0049 (PMC8896219; doi:10.1089/whr.2021.0049)
Supplement: Supplemental data [file Suppl_AppTableS2.docx]

Table 2. Correlation Analyses^1^ for seven protective factors examined in the Pregnancy Outcomes and Community Health Study, 1998-2004 (n=2,474)^2^

| Variables | 1. | 2. | 3. | 4. | 5. | 6. | 7. |
| --- | --- | --- | --- | --- | --- | --- | --- |
| 1. Self-esteem | -- | -- | -- | -- | -- | -- | -- |
| 2. Mastery | 0.48** | -- | -- | -- | -- | -- | -- |
| 3. Religiosity | 0.02 | 0.05* | -- | -- | -- | -- | -- |
| 4. Perceived Social Support | 0.27** | 0.32** | 0.02 | -- | -- | -- | -- |
| 5. Emotional social support | 0.23** | 0.19** | 0.02 | 0.27** | -- | -- | -- |
| 6. Instrumental Social support | 0.29** | 0.27** | 0.02 | 0.36** | 0.37** | -- | -- |
| 7. Reciprocity | 0.12** | 0.12** | 0.08** | 0.12** | 0.08** | 0.14** | -- |

*p<0.05 **p<0.01

^1^Standardized coefficient estimates for survey weighted regression procedures

^2^Results are weighted to account for the stratified sampling structure of the POUCH study
