# Supplementary material for: Differences in Psychosocial Protective Factors by Race/Ethnicity and Socioeconomic Status and Their Relationship to Preterm Delivery
Source: Womens Health Rep (New Rochelle). 2022 Feb 28;3(1):243–55. doi: 10.1089/whr.2021.0049 (PMC8896219; doi:10.1089/whr.2021.0049)
Supplement: Supplemental data [file Suppl_AppTableS3.docx]

Table 3. Associations between seven protective factors and odds of preterm delivery (PTD) assessed in logistic regression models with all protective factors included together in the Pregnancy Outcomes and Community Health Study, 1998-2004 (n=2,474)^1^

|  |  | Model 1^2^ |  | Model 2^3^ |  |
| --- | --- | --- | --- | --- | --- |
| Protective Factors |  | OR (95% CI) | | OR (95% CI) |  |
| *Individual-level* |  |  |  |  |  |
| Self-esteem |  | 1.1 (1.0, 1.2) |  | 1.1 (1.0, 1.2) |  |
| Mastery |  | 1.0 (0.9, 1.0) |  | 1.0 (0.9, 1.0) |  |
| Religiosity |  | 1.0 (0.7, 1.3) |  | 1.0 (0.7, 1.3) |  |
| *Interpersonal-level* |  |  |  |  |  |
| Perceived Social  Support |  | 1.0 (0.9, 1.1) |  | 1.0 (0.9, 1.1) |  |
| Emotional Social  Support |  | 0.6 (0.3, 1.3) |  | 0.6 (0.3, 1.2) |  |
| Instrumental  Social  Support |  | 1.1 (0.8, 1.3) |  | 1.1 (0.9, 1.5) |  |
| *Neighborhood-level* |  |  |  |  |  |
| Reciprocity |  | 1.0 (0.9, 1.0) |  | 1.0 (0.9, 1.0) |  |

**OR** odds ratio

^1^Results are weighted to account for the stratified sampling structure of the POUCH study; For continuous variables (self-esteem, mastery, perceived social support, instrumental social support, reciprocity) the OR represents the odds of PTD among women who have higher levels of the protective factor compared to women who have lower levels. For categorical variables (religiosity and emotional social support), the OR represents the odds of PTD among women who responded “yes” compared to women who responded “no.”

^2^Model 1: unadjusted with protective factors assessed together

^3^Model 2: Model 1 + race/ethnicity, SES, maternal age, parity, and marital status
